# Effects of a Media Prevention Program on Media-Related Knowledge and Awareness in Children and Their Parents: A Non-Randomized Controlled Cluster Study

**DOI:** 10.3390/pediatric18010004

**Published:** 2025-12-25

**Authors:** Tanja Poulain, Wieland Kiess, Team Drahtseil, Christof Meigen

**Affiliations:** 1 LIFE Leipzig Research Center for Civilization Diseases, Faculty of Medicine, Leipzig University, 04103 Leipzig, Germany; 2 Department of Women and Child Health, Hospital for Children and Adolescents and Center for Paediatric Research (CPL), Leipzig University, 04103 Leipzig, Germany; 3 German Center for Child and Adolescent Health (DZKJ), Partner Site Leipzig/Dresden, 04103 Leipzig, Germany; 4 Diakonisches Werk Innere Mission Leipzig e.V., Drahtseil, 04105 Leipzig, Germany

**Keywords:** media use, children, prevention program, school

## Abstract

Background/Objectives: This study evaluates a media prevention program conducted in elementary schools. Methods: A one-week media program, carried out with fourth graders in Leipzig, Germany, was evaluated using a non-randomized controlled cluster design. Program participants (experimental group (EG), *n* = 84 children, 41 parents) and non-participants (control group (CG), *n* = 19 children, 14 parents) completed questionnaires before the media program (t1), directly after the program (t2, EG only), and 3 months later (t3). The child questionnaire assessed media use frequency, rules at home, perceived and objective media-knowledge, and awareness of dangers on the Internet. The parent questionnaire assessed media-related topics discussed with their children and parents’ confidence regarding media education. Results: In the EG, children’s perceived and objective knowledge and their awareness of dangers on the Internet increased significantly between t1 and t2 and remained stable until t3, while no changes were observed in the CG. The number of children reporting that there exist rules on social media use also increased significantly in the EG but not in the CG. The amount of media-related topics discussed within the family and parents’ confidence regarding media education increased significantly from t1 to t3 in both EG and CG. Children’s media use frequency did not change across time, neither in the EG nor in the CG. Conclusions: Media prevention programs at school can have positive effects on children’s knowledge and awareness of dangers on the Internet and might improve parents’ confidence in and the realization of media education at home.

## 1. Introduction

The use of electronic media in children and young adolescents has increased considerably in recent decades [1]. In Germany, primary school age (roughly 6 to 10 years) is of particular importance with regard to the use of electronic media. At that age, children often start owning their own smartphones and tablets, usually accompanied by the first use of social media [2]. Playing video games also becomes more and more popular at this age [2]. Despite several advantages of the use of electronic media, e.g., fun, social exchange, and search for information, intensive use also bears some risks, e.g., negative consequences on mental health [3,4,5,6,7], sleep [8,9,10], and academic performance [11,12].

Unfortunately, children starting using electronic media are not always informed about the challenges and potential risks of use (e.g., data privacy, violence in video games), e.g., by parents or teachers. Active mediation (e.g., talking about media content) is assumed to be an effective media regulation strategy of parents [13,14]. However, this strategy requires time and knowledge of the latest media trends, of potential dangers, and of ways of counteracting them. The regulatory strategy applied most frequently by parents in Europe is time restriction [15,16], which has been shown to be less effective than active mediation [13].

An early exposure to electronic media is associated with a higher risk to show a problematic use of electronic media (especially the Internet) [17,18]. Problematic media use is characterized by excessive use that interferes with children’s functioning [19]. In addition to poor adult support, a lack of awareness, poor judgment, and the lack of knowledge about potential dangers of media use in younger children might be possible reasons for this observation.

One possibility to promote media competence in young children is prevention programs conducted at school. Programs conducted at school have the advantage that a large number of children can be reached. Also, children from families with a low socio-economic status, a known risk factor for child media use [20] but also for study recruitment and retention [21], can be reached more easily within the school context. In addition, prevention programs conducted at school might not only have a positive effect on child behavior or attitudes related to the content of the program (e.g., media use) but also on the class climate and inter-personal relationships.

Many different media prevention programs are offered at schools. However, evaluations on the (long-term) effects of these programs are sparse [22,23,24], also in Germany [25,26]. Of the evaluated programs, most focused on older age groups, e.g., adolescents, and did not include parental involvement [24]. A main finding of a recent systematic review on media literacy programs in schools is that programs are more helpful when the participating children are younger, when the program runs over a longer period of time or includes many sessions and when it contains interactive and practical elements [22]. A meta-analysis further indicated that programs involving not only children/adolescents but also their parents were especially effective [27].

In our study, we evaluated a media prevention program for 9- to 10-year-old children (i.e., children at primary school) that extends over 5 school days and includes several interaction and practical parts. We assessed whether or not participation in the media program was associated with changes regarding media use frequency, objective media-related knowledge, perceived media-related knowledge, and awareness of dangers on the Internet. As parents could partly be involved in the media program, we also assessed effects on parents’ confidence regarding media education and on media-related topics discussed at home. We expected that children participating in the program would show higher objective and perceived media-related knowledge as well as higher awareness after participation than before participation. In parents of participating children, we hypothesized to see increases in confidence and in the number of media-related topics discussed with the child. Regarding media use frequency, in contrast, we expected no positive effects of the program.

## 2. Materials and Methods

### 2.1. Design and Participants

**Figure 1:**
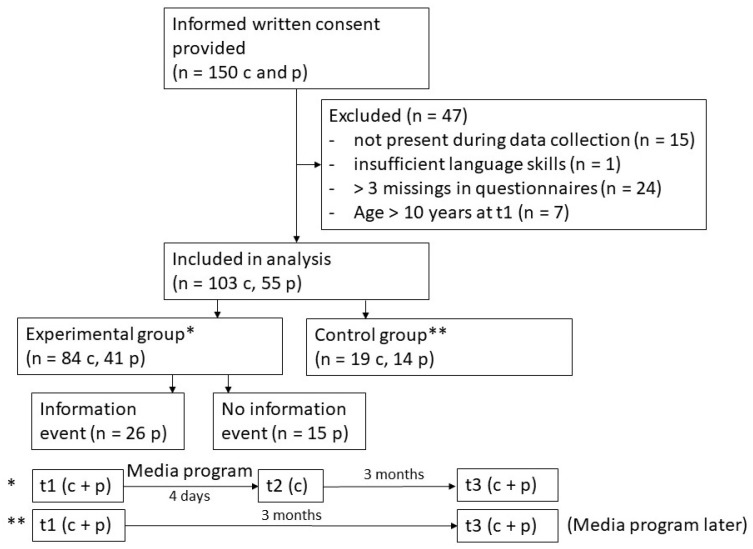
Study design and assignment to experimental and control group. c: children, p: parents.

The evaluation study was designed as a non-randomized controlled cluster trial. The media program to be evaluated was conducted in school year 2023/2024 in grades 4 (age 9–10 years) of four different primary schools in Leipzig, Germany (schools A–D). All children in the participating classes (three classes in schools A, B, and D, one class in school C) whose parents had provided informed written consent were included in the evaluation study (*n* = 150, 68% of all children participating in the program, see Figure 1). Of these children, 47 children were excluded due to different reasons (not present when the program was performed (*n* = 15), poor knowledge of German (*n* = 1), more than three missing answers in at least one questionnaire (*n* = 24), and age > 10 (*n* = 7)). The final child sample comprised 103 9- to 10-year-old children from schools A (*n* = 32), B (*n* = 21), C (*n* = 12) and D (*n* = 38). Of the participating parents, 55 (53%) completed the questionnaire at all time points and, thus, were included in the analyses.

To evaluate the quality of the media program, we compared an experimental group (EG) and a control group (CG). In the EG, participating children completed questionnaires on their media use, their media knowledge, their media-related attitudes, and their perception of class climate directly before the media program (t1), directly after the media program (t2), and three months later (t3), as shown at the bottom of Figure 1. Their parents were invited to attend an information event for parents that took place around the same time as the media program at school. They completed questionnaires on their own media use and their confidence regarding media education at t1 and t3. All classes of schools A, B, and C and one class of school D belonged to the EG (*n* = 84 children and 41 parents, see Figure 1). Within the EG, 26 parents (63%) attended the information event (EG-info), while 15 (37%) did not (EG-simple).

In the control group (CG), children and their parents completed the same questionnaires as the EG at t1 and three months later (t3), as shown at the bottom of Figure 1. However, in the CG, the media program, including the information event for parents, only started after t3. The CG comprised two classes at school D (*n* = 19 children and 14 parents, see Figure 1). In schools A, B, and C, it was not possible (for logistic reasons) that individual classes completed the media program 3–4 months later than other classes. Therefore, none of the children in these schools could be assigned to the CG.

Informed written consent was provided by all parents prior to the inclusion of their children in the study. The study was designed in accordance with the Declaration of Helsinki, and the study design was approved by the Ethics Committee of the Medical Faculty of Medicine at Leipzig University (381/22-ek, 8 November 2022).

### 2.2. Media Program

The media program (called “Medienführerschein” (Media License)) is a five-day interactive program (five hours per day) in which children are introduced to topics such as safety on the Internet, age restrictions for computer games and movies, advertising strategies, smartphone settings, and potential dangers of social media. It was designed in 2017 and is regularly adapted by the staff of the project Drahtseil. Drahtseil is a project that designs and conducts different prevention programs on media use, violence, and addiction for children in the city of Leipzig, Germany. Target audience of the media prevention program are children aged 9 to 10 years in the last year of primary school. During the whole program, two social workers and some student apprentices work with 15–25 children of the same class. It is conducted in the classroom of the pupils. The program exists of discussion rounds, interactive games, group work, and tasks for at home (e.g., discussing with parents, trying to find alternatives to media use at home). On the first day, children learn something about electronic devices in general and about advertising strategies. On the second day, they speak about video games and related age restrictions. On the third day, they discuss the internet, especially data protection, secure passwords, and search engines for children. The main topic of the fourth day is the mobile phone or smartphone. Children learn something about smartphone settings, social media, online bullying and child grooming. During the whole week, pupils also create an own interactive media project, e.g., a stop motion movie, comic, or podcast. On the last day, they present what they have learned in the week. Parents are invited to attend this presentation.

Within the media program, there is also an information evening for parents and/or guardians of the children, which takes place at school. It takes about two hours, during which parents are informed about ways to limit children’s screen time and to make it safer, ways to seek help with problematic use, and online bullying and child grooming.

### 2.3. Evaluation Questionnaires

The questionnaires were designed by our research team. First drafts of the questionnaires were evaluated by the educational staff of the Drahtseil team and tested on a small sample of children aged 9 years and their parents (*n* = 5 parent–child dyads). After this test phase, the questionnaires were shortened slightly.

The child participants completed questionnaires at t1, t2, and t3 at school. While the children completed the questionnaire, the Drahtseil staff made sure that the children do not exchange information and do not copy from each other. Parents completed questionnaires at t1 and t3. They could either complete a paper-pencil version of the questionnaire at home and send it back to school or complete the questionnaire online. Of the 55 parents completing questionnaires at both time points, about half completed the questionnaire online (47% at t1, 60% at t3). Table 1 (children) and Table 2 (parents) summarize the questionnaire items (including response options), the time point of assessment, and the resulting variables used for statistical analysis.

### 2.4. Statistical Analysis

For the descriptive analysis, we analyzed data collected at t1. The variables were described in terms of means and standard deviations (for numbers and sum scores), or frequencies and percentages (for categorical and dichotomous variables).

For the evaluation analysis, we compared variables collected at t1, t2, and t3 in both EG and CG (time point as independent variable). In addition, we compared variables between EG and CG at t1 and t3 (group as independent variable).

All data were analyzed using R version 4.3.1 [28]. To account for the hierarchical structure of the data, we fitted mixed-effects models. Children were nested within schools; therefore, random intercepts for school were included in all models to account for clustering at the school level. For analyses including repeated measurements, random intercepts for child ID were additionally specified to account for within-child dependencies across time points. Linear mixed-effects models were estimated using restricted maximum likelihood, whereas generalized linear mixed-effects models were estimated using maximum likelihood. Child age and sex were included as covariates in all models. Analyses on differences between EG and CG at t3 were additionally adjusted for the assessed variables at t1. An association or difference was considered statistically significant if *p* was <0.05.

Missing information in more than three variables at one time point led to the exclusion of the child of the whole study. The remaining missing values (maximum of three per time point) led to the exclusion of the child in the specific analysis only.

## 3. Results

### 3.1. Description

The distributions of the variables analyzed in the study are shown in Table 3 (children) and Table 4 (parents), separated by group (EG versus CG).

Within the child sample (*n* = 102), 10-year-olds (about 60%) and girls (about 60%) were overrepresented compared to 9-year-olds and boys. The media device owned most frequently was a game console (about 70%), followed by a smartphone (about 60%). Concerning media use frequency, about half of all children reported to watch movies/series and/or to play video games every day. The use of social media was less frequent.

The parents’ questionnaires were most frequently completed by mothers (about 80%). Education of the participating parents was high, with about 60% reporting the highest school degree. About 75% of all parents used social media every day, while watching movies/series and playing video games were less popular.

T-tests and Chi-squared tests showed that EG and CG did not differ significantly regarding child age, sex of children and parents, SES of parents, and media use frequency of parents (all *p* > 0.05). Differences in the other variables are mentioned in the paragraphs on effects of the media program.

### 3.2. Effects of the Media Prevention Program in Children

Differences between EG and CG at t1 and t3 regarding perceived knowledge, objective knowledge, and awareness as well as differences between t1, (t2), and t3 in both groups are shown in Table 5.

**Figure 2:**
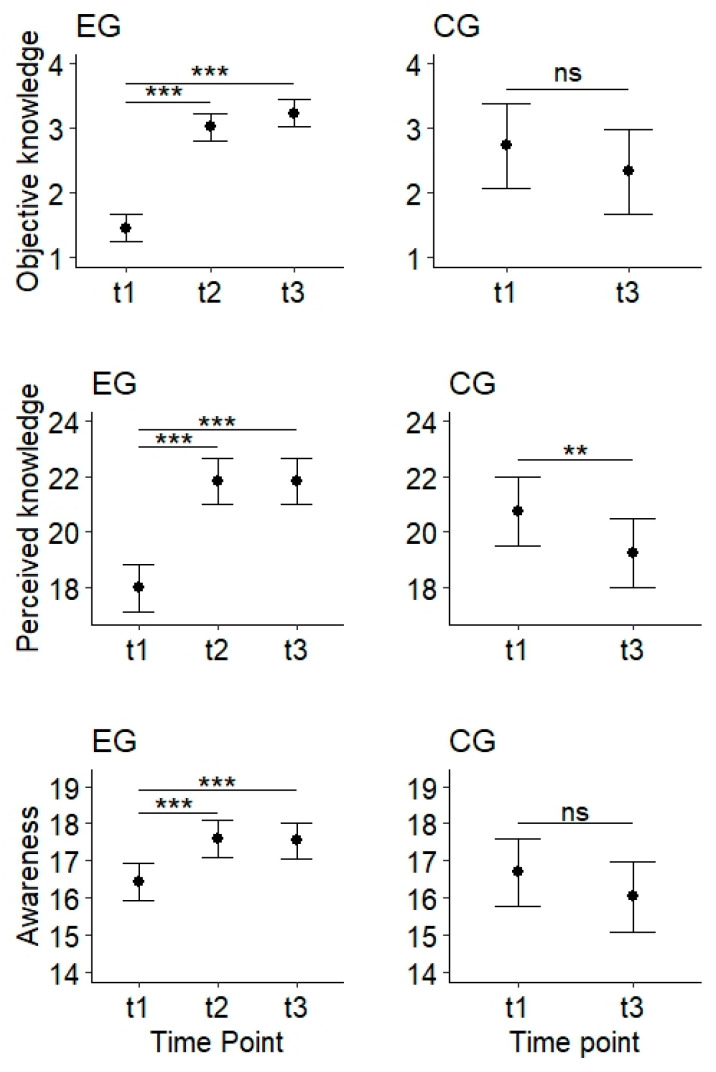
Objective knowledge (number of correct answers, possible range 0–4), perceived knowledge (score, possible range 6–24), and awareness (score, possible range 5–20) at the different time points in the children’s experimental group (EG, left, *n* = 84) and the control group (CG, right, *n* = 19). t1: directly before the media program, t2: directly after the media program, t3: three months after the media program. *** *p* < 0.001, ** *p* < 0.01, ns: not statistically significant.

Concerning children’s objective knowledge (see Figure 2), we observed an unexpected difference between EG and CG at t1, with significantly more correct responses in the CG. As expected, in the EG, responses at t2 and t3 were more frequently correct than at t1, with no significant difference between t2 and t3. In the CG, in contrast, we observed no significant change between t1 and t3. Finally, at t3, children of the EG gave correct answers significantly more frequently than children of the CG.

Regarding children’s perceived knowledge (see Figure 2), we observed no significant difference between EG and CG at t1. In the EG, perceived knowledge increased significantly from t1 to t2, as hypothesized. At t3, it was still significantly higher than at t1, and similar to the perceived knowledge at t2. Surprisingly, in the CG, perceived knowledge decreased significantly between t1 and t3. At t3, perceived knowledge in the CG was significantly lower than in the EG, as expected.

With respect to children’s awareness (see Figure 2), the analyses were in line with our hypotheses. At t1, awareness in EG and CG did not differ significantly. While awareness in the EG increased significantly from t1 to t2 and remained stable between t2 and t3, no significant change was observed in the CG. At t3, awareness was significantly higher in the EG than in the CG.

With respect to media use of children (only assessed at t1 and t3), we observed no significant differences between EG and CG regarding the number of devices, the frequency of use, and media regulation at home, neither at t1 nor at t3. However, while the number of media devices owned by the child increased significantly from t1 to t3 in the CG, no significant change was observed in the EG. With respect to the frequency of use, the analyses revealed no change between t1 and t3, neither in the EG nor in the CG. Concerning media regulation at home, we observed a significant increase in rules regarding social media from t1 to t3 in the EG, but not in the CG. Rules regarding movies/series and video gaming, in contrast, did not change significantly between t1 and t3, neither in the EG nor in the CG.

In the EG, we also checked changes in the perceived class climate and children’s appreciation of group work between t1 and t2. Both the perception of class climate as “very good” and the “very high” appreciation of group work increased significantly between t1 and t2.

Within the media program, children of the EG were also asked to create a “media use contract” with their parents, in which the framework of media use is set out (e.g., time limits, media-free times for both child and parents). At t3, 66 children of the EG (80%) reported having created such a contract with their parents (compared to 0% in the CG). Of these children, 91% (*n* = 60) reported that the family adheres completely (*n* = 18, 27%) or at least partly (*n* = 42, 64%) to the rules laid down in the contract. Only 9% (*n* = 6) reported that the family does not adhere to it.

### 3.3. Effects of the Media Program in Parents

Table 6 shows the differences between EG-info, EG-simple and CG at t1 and t3 regarding the number of aspects discussed with the child and confidence, as well as differences between t1 and t3 in all groups.

**Figure 3:**
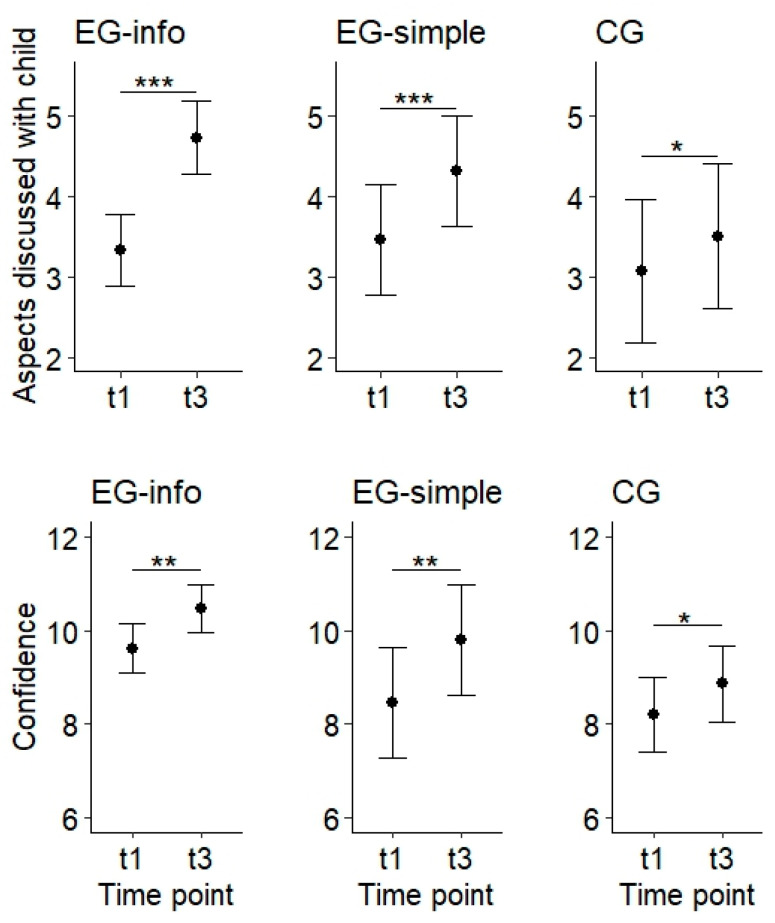
Aspects discussed with child (sum, possible range 0–5) and confidence (score, possible range 3–12) at the different time points in the parents’ experimental group attending the information event (EG-info, left, *n* = 26), the experimental group not attending the information event (EG-simple, middle, *n* = 15) and the control group (CG, right, *n* = 14). Legend: t1: directly before the media program, t3: three months after the media program. *** *p* < 0.001, ** *p* < 0.01, * *p* < 0.05.

Regarding the number of aspects already discussed with the child, we observed no significant differences between groups at t1. In all three groups, the number of aspects discussed increased significantly between t1 and t3 (see Figure 3). As revealed by a supplemental analysis, this increase was significantly stronger in parents of the EG who also attended the information event compared to parents of the CG (b = 0.96 (95% CI 0.32, 1.61), *p* = 0.005). At t3, parents of both EG (with and without having attended the information event) had talked about significantly more aspects of media use/internet than parents of the CG.

Similarly to the aspects discussed with the child, the confidence score of parents at t1 did not differ between groups. Also, the confidence score increased significantly between t1 and t3 in all groups (see Figure 3). The strength of this increase did not differ significantly between groups, as revealed by a supplemental analysis. At t3, confidence was significantly higher in both EG (with and without having attended the information event) than in the CG.

## 4. Discussion

The present study investigated the effects of a media prevention program conducted in 9- to 10-year-old children. The descriptive analysis showed that game consoles and smartphones are the devices that children owned most frequently. Watching movies/series and playing video games were the most frequent media activities. Compared to the results of a large survey of 6- to 13-year-old children in Germany from 2024, children in our study reported a higher frequency of gaming and watching movies/series [2]. In parents, the use of social media was more frequent than watching movies/series or playing video games.

While more than half of the children reported that there exist rules regarding watching movies/series and gaming, rules regarding social media use were less frequent. A reason for this finding might be that social media are often used on the smartphone, i.e., a mobile device that might also be used in places other than at home and whose use, therefore, might be more difficult to control [29].

### 4.1. Effects of the Media Prevention Program in Children

With regard to the main question of the effects of the media prevention program on children’s knowledge and media use, our hypotheses could largely be confirmed. With one exception—higher objective knowledge among children in the CG compared to the EG—the variables assessed at t1, including the distributions of age, sex, and SES, were not significantly different between CG and EG. Therefore, although the CG was much smaller and more homogeneous than the EG, overall, both groups can be considered comparable before the media program was implemented.

It is difficult to explain why objective knowledge at t1 was better in the CG than in the EG (and also than in the CG 3 months later, although not significantly). One potential explanation is that the media knowledge at t1 was actually higher in the CG than in the EG, e.g., because children of these particular classes (the CG only comprised two classes) had already discussed this topic more intensively than children in the other classes. In this case, however, one would not have expected media knowledge to decline within three months. Another explanation could be that some of the children in the CG helped each other. The staff actually ensured that children were not able to speak with each other or to copy from others. However, one might speculate that in this case (t1 in the CG), this could not be completely prevented.

As expected, we observed a significant improvement in perceived and objective knowledge and awareness in the EG from before participation to after participation in the media program, which could still be observed three months later. In the CG, no change was observed over time (perceived knowledge even became significantly worse). At t3, children in the EG performed significantly better than children in the CG in all three areas. These differences should be interpreted with caution, given the small and homogeneous CG. However, they suggest that the media program in the last year of elementary school has led to an increase in objective and perceived knowledge and awareness. Overall, these positive effects are in line with systematic reviews in which several of the reviewed studies reported positive effects of school-based media programs on outcomes related to knowledge, understanding, empowerment, and awareness [22,23,24].

With regard to media use frequency, the effects were far less pronounced, as expected. The frequency of use of electronic devices did not change over time in either the EG or the CG. This finding is partly in line with findings of another evaluation study conducted in Germany. In that study, a media program showed a positive effect on gaming time but not on the time spent using the Internet [25]. Established media usage times are often firmly integrated in children’s everyday lives (e.g., through times set by parents) and, therefore, might be difficult to change through prevention programs. However, it is important to note that the main aim of the media prevention program was not to reduce usage times but to strengthen awareness and increase knowledge and safety.

The frequency of rules regarding the use of TV and video games also did not change over time in any group. Interestingly, however, there was an increase in the frequency of rules regarding the use of social media in the EG (not in the CG). The use of social media is less frequent at primary school age and is associated with more uncertainty than the use of television and video games. Possible dangers and the importance of rules play an important role in the media prevention program and in the information event for parents. This might explain why there has been an increase in corresponding rules in the EG. However, it should be noted that the effect found was very small.

A similarly small, but significant effect was seen with regard to the number of media devices. We observed an increase from before to three months after the media program in the CG, but not in the EG. Participation in the media program might have encouraged EG children not to seek for additional devices and/or their parents to not buy additional devices. Also, as the CG only included children of two classes, it is possible that specific media devices were especially popular in these classes and, therefore, were purchased more frequently in these children. Overall, however this specific result should be interpreted with caution. The CG was very small, and the purchase of media devices is a very personal matter that is influenced by many factors, including income, education [30], or presence or age of (older) siblings.

For a better classification of the results regarding rules and media devices, it would have been useful to have information about whether or not the children’s parents attended the information event. Unfortunately, this information was only available for children whose parents had completed the parent’s questionnaire at t3 (53%) and, thus, could not be considered in the child analyses.

The results of the study show that the media program may also have positive effects on the perceived classroom climate and motivation to work together. In the EG, both aspects were rated significantly better after participation in the media program than before participation. This finding underlines findings of a previous study in which an active learning intervention had positive effects on classroom climate in college students [31]. In our study, we did not investigate the extent to which these improvements can also be observed in the long term. However, the results indicate that programs outside of the normal school curriculum are perceived as pleasant and can improve the climate in the classroom. This is probably due to the interactive parts of the program and working without pressure to perform.

### 4.2. Effects of the Media Prevention Program in Parents

Just over half of the parents of the children participating in the study (49% of the EG and 74% of the CG) completed the parental questionnaires. This illustrates the difficulty of motivating parents to complete questionnaires. The offer to complete the questionnaires online was used by about half of the parents. Nevertheless, this option did not increase the overall participation rate to over 60%.

Within the sample of participating parents, the large majority was female. The fact that fathers are less involved in research in children is well known. They also participate less frequently in prevention or intervention programs [32]. Due to the small number of parents surveyed, the effects shown here should be interpreted with caution.

As the parents’ questionnaire asked whether they had attended the information event, we were able to distinguish two groups within the parents’ EG: parents who had attended the information event and parents who had not. Fortunately, the proportion of parents who attended the information event was very high (63%).

Prior to the children’s participation in the media program, the three groups of parents (CG, EG attending the information event, and EG not attending the information event) did not differ significantly in terms of media use frequency, the number of media topics discussed with the child and the perceived confidence. Between t1 and t3, the number of media topics discussed and the confidence increased significantly in all groups. This could indicate that the end of primary school is generally a time when parents discuss media-relevant topics with their children. After fourth grade, children in Germany transition to secondary school. From fifth grade onwards, children are expected to be more independent. Therefore, the topic of media use (e.g., whether children should have their own smartphones or be allowed to use the internet without parental supervision) could become increasingly important for both children and parents during the fourth grade. Parents may also seek information on this topic outside of the school context and thus gain confidence.

Interestingly, the increase in the number of topics discussed with the child was significantly stronger in the EG that had attended the information event than in the CG. The information event might encourage parents even more to talk about media topics. The finding that 3 months after the media program, parents of both experimental groups (with or without attended the information event) had discussed significantly more topics and had significantly higher confidence scores than parents of the CG also suggests that the media program had a positive effect in parents.

### 4.3. Strengths and Limitations

Strengths of the study are the comparison of a control and an experimental group, the young age of the participating children, the investigation of different outcomes at three different time points, and the inclusion of parents’ perspectives.

A major limitation is the small sample size, especially regarding children in the control group. This reduces the statistical power in the control group, which may have prevented potential effects from being detected. Furthermore, the control group was very homogeneous because it only included children from two school classes. Therefore, experimental and control group probably already differed before the intervention, and observed differences might be attributed not only to the media program but also to these pre-existing differences. This limits the internal validity of the study.

As standard questionnaires often exist only for older children or are very long, we relied on questions created within our research team. These questionnaires were not validated previously. Therefore, it is not clear whether they really recorded what they were supposed to record.

The lack of information on parents’ education is another limitation. This information was only available for parents who had completed the parental questionnaire.

Finally, given the small number of clusters in this study, standard-errors and test statistics might be unstable, which may result in lower reliability of precision and less robust conclusions.

## 5. Conclusions

The 5-day media prevention program, which was carried out with 9- to 10-year-old pupils at the end of elementary school, shows positive effects on awareness, perceived and objective knowledge in participating children, even after 3 months. The information event for parents stimulates discussions about media-related topics with the children. Media usage times, on the other hand, cannot be changed by the media program, and effects on media rules at home and the ownership or purchase of new devices are not clear.

## Figures and Tables

**Table 1:** Questions answered by children at different time points, and the resulting variables used for statistical analysis.

Time	Topic	Content	Variable(s)
t1, t3	Device ownership	Ownership of 5 devices (TV, smartphone, smartwatch, laptop/tablet, game console) Response options: yes; no	Number of own devices (range 0–5)
t1, t3	Media use frequency	Usage frequency for 3 media activities (movies/series, video games, social media) Response options: (nearly) each day, ≥2 h (4); (nearly) each day, <2 h (3); 3–4 times/week (2); 1–2 times/week (1); less frequently or never (0)	Dichotomous: daily versus not daily, for each media activity; sum score (range 0–12, higher scores indicate more frequent use)
t1, t3	Rules	Rules at home (movies/series, video games, social media) Response options: yes; no	Dichotomous: yes versus no, for each media activity
t3	Media use contract	Media use contract Response options: yes; no	Dichotomous: yes versus no
t1, t2, t3	Objective knowledge	4 questions on media knowledge: 1. naming two components of secure passwords (open), 2. naming three behaviors that help in case of harassment on the Internet (open), 3. naming a search engine for children on the Internet (open), 4. naming who is responsible for photos on the Internet (response options: person on the photo (=correct); person taking the photo; person sending the photo)	Number of correct responses (range 0–4)
t1, t2, t3	Perceived knowledge	Knowledge on 6 aspects of media use/Internet (age restrictions, online privacy, password security, online bullying, online search strategies, social media) Response options: not at all (1); rather no (2); rather yes (3); yes (4)	Sum score (range 6–24, higher scores indicate higher self-rated knowledge) ^a^
t1, t2, t3	Awareness	Awareness regarding 5 aspects of media use/Internet (photos on the Internet, trustworthiness of the Internet, online contact to strangers, cluelessness on the Internet, sharing personal data) Response options: not at all (1); rather no (2); rather yes (3); yes (4)	Sum score (range 5–20, higher scores indicate higher awareness) ^b^
t1, t2 EG only	Group work	Pleasantness of group work Response options: very pleasant; rather pleasant; rather not pleasant; not pleasant at all	Dichotomous: very pleasant versus lower
t1, t2 EG only	Class climate	Satisfaction with the class mates Response options: very low; rather low; rather good; very good	Dichotomous: very good versus lower

^a^ internal consistency: alpha = 0.76. ^b^ internal consistency: alpha = 0.70.

**Table 2:** Questions answered by parents at different time points, and the resulting variables used for statistical analysis.

Time	Topic	Content	Variable(s)
t1	Education	Highest school degree Response options: no school degree; lowest secondary school; middle secondary school; highest secondary school	Dichotomous: high versus low/middle (“no school degree” was not reported)
t1	Media use frequency	Usage time for 3 media activities (movies/series, video games, social media) Response options: (nearly) each day, ≥3 h (4); (nearly) each day, <3 h (3); 3–4 times/week (2); 1–2 times/week (1); less frequently or never (0)	Dichotomous: daily versus not daily, for each media activity; sum score (range 0–12, higher scores indicate more frequent use)
t1, t2	Discussion with child	Discussion on 5 topics (age restriction, online bullying, online shopping, fake news, online privacy) Response options: yes; no	Number of topics already discussed (range 0–5)
t1, t2	Confidence	Confidence regarding 3 subjects (media education, finding information regarding media education, applications for secure use) Response options: not at all (1); rather not (2); rather yes (3); totally (4)	Sum score (range 3–12, higher scores indicate more confidence) ^a^

^a^ internal consistency: alpha = 0.77.

**Table 3:** Distribution of assessed variables in participating children (*n* = 103), separated by group (experimental (EG) and control (CG)).

		T1		T2	T3	
		EG (*n* = 84)	CG (*n* = 19)	EG (*n* = 84)	EG (*n* = 84)	CG (*n* = 19)
Age	*n* (%) 9 years	30 (36%)	8 (42%)	-	14 (17%)	6 (32%)
	*n* (%) 10 years	54 (64%)	11 (58%)	-	70 (83%)	13 (68%)
Sex	*n* (%) male	37 (44%)	6 (32%)	-	-	-
	*n* (%) female	47 (56%)	13 (68%)	-	-	-
Device ownership						
TV ^a^	*n* (%)	18 (21%)	6 (32%)	-	16 (19%)	6 (32%)
Smartphone ^a^	*n* (%)	49 (59%)	12 (63%)	-	60 (71%)	14 (74%)
Smartwatch ^a^	*n* (%)	21 (25%)	4 (21%)	-	24 (25%)	4 (21%)
Laptop/Tablet ^a^	*n* (%)	51 (61%)	5 (28%)	-	56 (67%)	8 (42%)
Game console ^a^	*n* (%)	58 (71%)	11 (68%)	-	62 (75%)	13 (68%)
Number of media devices	Mean (sd)	2.4 (1.2)	2.0 (1.2)	-	2.6 (1.3)	2.4 (1.2)
Media use frequency						
Movies/series ^a^	*n* (%) daily use	47 (56%)	11 (58%)		43 (51%)	11 (58%)
Video games ^a^	*n* (%) daily use	37 (45%)	8 (42%)		45 (54%)	6 (32%)
Social media ^a^	*n* (%) daily use	32 (38%)	5 (28%)		34 (41%)	7 (39%)
Frequency of use (score)	Mean (sd)	6.2 (3.3)	5.1 (3.0)	-	6.3 (2.9)	5.3 (2.4)
Media Rules ^b^						
Movies/series	*n* (%) yes	46 (66%)	8 (53%)	-	54 (77%)	10 (67%)
Video games	*n* (%) yes	49 (70%)	6 (50%)	-	50 (71%)	10 (77%)
Social media	*n* (%) yes	24 (50%)	3 (38%)	-	33 (69%)	4 (50%)
Media use contract	*n* (%) yes	-	-	-	66 (80%)	0 (0%)
Objective knowledge						
Number correct	Mean (sd)	1.4 (1.1)	2.7 (1.2)	3.0 (1.0)	3.2 (0.8)	2.3 (1.2)
Self-rated knowledge						
Sum score	Mean (sd)	18.0 (4.1)	20.7 (2.7)	21.8 (2.6)	21.8 (2.2)	19.2 (2.5)
Awareness						
Sum score	Mean (sd)	16.5 (2.4)	16.7 (1.9)	17.6 (1.9)	17.6 (1.6)	16.0 (1.9)
Class climate						
Group work	*n* (%) very pleasant	24 (29%)	-	37 (44%)	-	-
Class climate	*n* (%) very good	16 (19%)	-	26 (31%)	-	-

EG: experimental group, CG: control group. ^a^ in further analyses, not analyzed separately but only as part of the number of media devices variable or the frequency of use score. ^b^ only assessed in children pursuing the activity.

**Table 4:** Distribution of assessed variables in participating parents (*n* = 55), separated by group (experimental (EG) and control (CG)).

		T1			T3		
		EG-Info (*n* =26)	EG-Simple (*n* = 15)	CG (*n* = 14)	EG-Info (*n* =26)	EG-Simple (*n* = 15)	CG (*n* = 14)
Sex	*n* (%) female	20 (73%)	12 (80%)	12 (86%)	18 (69%)	11 (73%)	11 (79%)
	*n* (%) male	6 (23%)	3 (20%)	2 (14%)	8 (31%)	4 (27%)	3 (21%)
Education							
Low/Middle	*n* (%) yes	10 (38%)	6 (40%)	5 (38%)	-	-	-
Highest	*n* (%) yes	16 (62%)	9 (60%)	8 (62%)	-	-	-
Media use frequency							
Movies/series ^a^	*n* (%) daily use	11 (42%)	7 (47%)	4 (29%)	-	-	-
Video game ^a^	*n* (%) daily use	4 (15%)	5 (33%)	3 (21%)	-	-	-
Social media ^a^	*n* (%) daily use	18 (69%)	13 (87%)	10 (71%)	-	-	-
Frequency of use (score)	Mean (sd)	5.2 (2.9)	6.2 (2.0)	4.7 (2.4)	-	-	-
Discussion with child							
Number topics	Mean (sd)	3.3 (1.5)	3.5 (0.9)	3.1 (1.5)	4.8 (0.7)	4.4 (0.5)	3.5 (1.6)
Confidence							
Sum score	Mean (sd)	9.6 (1.4)	8.7 (1.6)	8.2 (1.3)	10.5 (1.3)	10.0 (1.4)	8.9 (1.3)

EG-info: experimental group attending the information event for parents, EG-simple: experimental group not attending the information event for children, CG: control group. ^a^ in further analyses, not analyzed separately but only as part of the frequency of use score.

**Table 5:** Effects of the media prevention program in children: Differences between EG (*n* = 84) and CG (*n* = 19) at different time points and changes in EG and CG across time.

Variable		Difference EG-CG ^1^		Differences Between Time Points			
				In EG			In CG
		t1	t3	t2-t1 ^2^	t3-t1 ^2^	t3-t2 ^3^	t3-t1 ^2^
Knowledge	b	−1.27	0.92	1.57	1.78	0.21	−0.40
	95% CI	−1.86, −0.69	0.36, 1.48	1.30, 1.85	1.50, 2.06	−0.07, 0.48	−1.08, 0.28
	*p*	0.001	0.002	<0.001	<0.001	0.143	0.271
Perceived knowledge	b	−2.03	3.11	3.89	3.89	0.00	−1.53
	95% CI	−4.26, 0.20	1.97, 4.24	3.15, 4.23	3.16, 4.63	−0.72, 0.73	−2.56, −0.49
	*p*	0.078	<0.001	<0.001	<0.001	0.994	0.010
Awareness	b	−0.10	1.65	1.17	1.11	−0.06	−0.66
	95% CI	−1.26, 1.06	0.77, 2.53	0.66, 1.69	0.60, 1.62	−0.56, 0.45	−1.61, 0.30
	*p*	0.865	0.003	<0.001	<0.001	0.823	0.196
Class climate	OR	-	-	2.49	-	-	-
	95% CI	-	-	1.01, 6.12	-	-	-
	*p*	-	-	0.046	-	-	-
Group work	OR	-	-	2.65	-	-	-
	95% CI	-	-	1.16, 6.03	-	-	-
	*p*	-	-	0.021	-	-	-
Number devices	b	0.40	−0.11	-	0.19	-	0.33
	95% CI	−0.29, 1.08	−0.63, 0.41	-	−0.02, 0.41	-	0.11, 0.56
	*p*	0.292	0.692	-	0.086	-	0.009
Frequency of use	b	0.61	0.51	-	0.08	-	−0.03
	95% CI	−10.34, 20.56	−0.67, 10.69	-	−0.53, 0.69	-	−0.98, 0.91
	*p*	0.538	0.399	-	0.794	-	0.949
Rules TV	OR	2.38	1.55	-	2.93	-	2.80
	95% CI	0.57, 9.99	0.36, 6.72	-	0.95, 9.08	-	0.32, 24.7
	*p*	0.235	0.558	-	0.062	-	0.352
Rules video games	OR	2.31	0.50	-	1.14	-	5.1
	95% CI	0.66, 8.06	0.09, 2.54	-	0.41, 3.09	-	0.65, 56.9
	*p*	0.189	0.405	-	0.799	-	0.141
Rules social media	OR	2.01	1.30	-	2.95		1.91
	95% CI	0.41, 9.90	0.24, 7.08	-	1.02, 8.55	-	0.19, 19.4
	*p*	0.391	0.764	-	0.047	-	0.583

EG: experimental group, CG: control group. ^1^ reference = CG, ^2^ reference = t1, ^3^ reference = t2.

**Table 6:** Effects of the media prevention program in parents: Differences between EG-info (*n* = 26), EG-simple (*n* = 15) and CG (*n* = 14) at different time points and changes in groups across time.

		Differences Between Groups ^1^				Differences Between t1 and t3 ^2^		
		t1		t3				
		EG-Info -CG	EG-Simple -CG	EG-Info -CG	EG-Simple -CG	EG-Info	EG-Simple	CG
Discussion with child	b	0.19	0.44	1.10	0.67	1.40	0.85	0.43
	95% CI	−0.73, 1.11	−0.58, 1.46	0.62, 1.57	0.13, 1.22	0.93, 1.86	0.42, 1.29	0.09, 0.77
	*p*	0.689	0.400	<0.001	0.019	<0.001	<0.001	0.028
Confidence	b	1.49	0.46	0.84	0.86	0.84	1.33	0.64
	95% CI	0.23, 2.76	−0.90, 1.83	0.10, 1.58	0.08, 1.64	0.36, 1.33	0.65, 2.01	0.08, 1.21
	*p*	0.052	0.527	0.032	0.037	0.002	0.002	0.045

EG-info: experimental group attending the information event for parents, EG-simple: experimental group not attending the information event for children, CG: control group. ^1^ reference = CG, ^2^ reference = t1.

## Data Availability

The legal requirements and the given informed consent do not allow public sharing of the dataset. Interested researchers can contact the research data management of the Medical Faculty, University Leipzig: forschungsdaten@medizin.uni-leipzig.de for further information. The dataset ID is PID-513.

## Abbreviations

The following abbreviations are used in this manuscript:

EG	experimental group
CG	control group
